# Breaking chains of tobacco: empowering African American churches in West Virginia for a healthier future

**DOI:** 10.3389/fpubh.2024.1472654

**Published:** 2024-09-19

**Authors:** Donald Reed, Truman Dangerfield, Rhonda Robinson, Kenneth Ray, Kathy Danberry, Kim Tieman

**Affiliations:** ^1^School of Health Sciences, Public and Community Health, Liberty University, Lynchburg, VA, United States; ^2^Marriott School of Business (Student), Brigham Young University, Provo, UT, United States; ^3^West Virginia African American Tobacco Prevention Network, Beckley, WV, United States; ^4^Center for Black Health and Equity, Atlanta, GA, United States; ^5^West Virginia Division of Tobacco Prevention, Charleston, WV, United States; ^6^Claude Worthington Benedum Foundation, Pittsburgh, PA, United States

**Keywords:** tobacco, menthol, cessation, African American, grassroots, network, rural

## Abstract

Across West Virginia, tobacco use continues to be a significant public health challenge. Specifically, tobacco use is linked to high poverty across the state and disproportionately affects African Americans. A faith-based tobacco prevention network was formed to address these concerns and increase education and cessation. The West Virginia African American Tobacco Prevention Network (WVAATPN) was formed in 2021 and since then has expanded its reach across the state, involving 22 congregations. The Network’s model includes annual training for lay leaders on various tobacco-related topics, tailored educational curriculum for congregations, and collaboration with national experts to enhance program efficacy. The Network has run educational and cessation workshops and promoted events such as No Menthol Sunday. Workshops have yielded positive outcomes among participants, including increased awareness of tobacco marketing tactics, higher cessation rates, and improved understanding of the health impacts of tobacco. The WVAATPN continues to expand its reach and effectiveness by advocating for policy change, enhancing community engagement, and fostering partnerships to combat tobacco-related disparities in West Virginia’s African American communities.

## 1 Background and rationale

Tobacco use remains a significant public health challenge in West Virginia, with high rates among adults and youth. According to recent data from the Campaign for Tobacco-Free Kids, 21% of West Virginia adults and 7.6% of high school students smoke cigarettes, and 27.5% of high schoolers use e-cigarettes. The impact is severe, with smoking contributing to 4,300 deaths annually and 37.8% of cancer deaths in the state (1). The absence of state-level Clean Indoor Air Regulations exacerbates the issue, with local regulations facing preemptive challenges (2, 3).

Part of the difficulty associated with the high tobacco usage rates in West Virginia stems from the extreme poverty across the state. Almost 18% of individuals in West Virginia live in poverty (4). Tobacco use and poverty are linked in a vicious cycle. Those living in poverty are often trapped into smoking for nearly twice as many years as those with higher incomes (5). Additionally, the health effects of tobacco use increase poverty, especially if the breadwinner becomes unable to work due to tobacco-related illness (6). This socio-economic factor intertwines with tobacco use in a cyclical pattern, as tobacco expenditure diminishes resources available for essential needs, exacerbating health disparities.

Despite the severe impact of tobacco use across the state, West Virginia has struggled to address the issue. While receiving over $227 million in Master Settlement Agreement funds and tobacco taxes in fiscal year 2024, the West Virginia Legislature only allocated $451,404 to tobacco prevention (1). West Virginia also received $1,083,616 in fiscal year 2023 from the Centers for Disease Control Office of Smoking and Health (7), and these two funding sources combined continue to fund the West Virginia Division of Tobacco Prevention. But West Virginia ranks number 50 in the US for tobacco prevention spending, spending less than 2% of the CDC recommendation for the state (1).

Due to limited tobacco-cessation funding across West Virginia, individuals who use tobacco face challenges in overcoming their addiction, as they lack adequate resources and support to quit (8). In addition, certain demographic groups are more likely to experience the health effects of tobacco use (5). Specifically, African Americans are targeted by tobacco companies through historical and ongoing marketing tactics, especially with menthol cigarettes (9). Despite having a similar cigarette use rate of 21.4%, African Americans are disproportionately affected by tobacco, which makes them a priority population for the West Virginia Division of Tobacco Prevention (10, 11). African Americans in West Virginia also have a higher poverty rate of 30.7% compared to the state average of 17.9% (4, 12). To combat the relentless marketing tobacco companies employ against African Americans, a plan was made to create a network of community organizations united in increasing education and cessation. A grassroots network was essential for combatting the deeply set cultural patterns of tobacco use among African Americans.

West Virginia has a population of 1.7 million, of which 3.8% identify as Black or African American, and 2.1% identify as Two or More Races (13). Due to West Virginia’s coal mining heritage, many African Americans live in population clusters: Bluefield, Beckley, Charleston, Martinsburg, Dunbar, Keyser, Charles Town, Oak Hill, South Charleston, and Huntington (14).

For generations, the tobacco industry has been targeting African Americans with its powerful marketing and segmented production decisions. It began in the 1950s when big tobacco companies decided to market menthol cigarettes to the growing African American market segment (15). They did this through traditional advertising and efforts to increase their credibility among African Americans. Phillip Gardiner stated:

Numerous social factors, when taken together, conspired to coerce the adoption of menthol cigarettes by a majority of African Americans in the 1960s and 1970s… Identification by young urban Blacks with menthols as “fresh and modern” helped establish these brands as an important part of the African American experience… At the same time that the industry vigorously pushed menthol products on Blacks, they also were giving money to Black community organizations, including civil rights groups. In essence, the tobacco industry successfully created an attachment to menthols that still resonates in the Black community today (16).

The promotional efforts and donations combined secured the tobacco industry’s foothold in the African American market, and in the 60 years since, they have not lost it. According to one study, data suggests that there are 2.6 times more tobacco-related advertisements in African American areas than in Caucasian regions (17). That is clear evidence of the relentless marketing big tobacco companies continue to use to target African Americans.

One outcome of their marketing was successfully branding menthol-flavored cigarettes as the “African American” cigarette. Due to its cool, soothing effect, menthol cigarettes reduce the irritation involved with smoking, which can make it easier to start and harder to quit (18). In 1971, only 38% of African American smokers used menthol cigarettes (16). After generations of menthol cigarette advertising targeted at African American adults, that number has risen to 80% (19).

In West Virginia, 26% of all adults who smoke use menthol cigarettes (20), and while menthol smoking rates for African Americans are unknown, we can assume they are comparable to the known national average of 80% (19). These and other factors made clear the need to focus on African Americans in developing the network.

## 2 Pedagogical framework

To combat the devasating effects of tobacco use among African Americans in West Virginia, Dr. Donald Reed desired to create a lay helper network of community organizations united in the cause to address tobacco use within African American churches. A previous study has shown that African American church coalitions could be effective in rural smoking interventions (21). Dr. Reed aimed to create a similar network of churches and community organizations.

Dr. Donald Reed is the Executive Director of the McDowell County Commission on Aging, Inc. and an Assistant Professor at Liberty University. He holds a doctorate in public health and is certified in public health by the National Board of Public Health Examiners and as a tobacco treatment specialist by the Mayo Clinic Nicotine Dependence Center. He has a 20-year career in public health service in the rural coalfields.

Dr. Reed’s plan for the network included a focus on unifying efforts to educate the community and provide evidence-based workshops to increase cessation. In addition, the network would provide the community with resources and connections to receive essential cessation and educational support. This grassroots approach would allow for greater cooperation among distinct organizations and greater dissemination of proven practices for reducing tobacco use. Specifically, the network was planned to target and support African Americans, who, while having similar tobacco usage rates, are disproportionately affected by tobacco use. African Americans are more likely to experience second-hand smoke and deaths caused by second-hand smoke have a disproportionate impact on African Americans (22, 23). The West Virginia Division of Tobacco Prevention believed in Dr. Reed’s vision and assisted in creating the West Virginia African American Tobacco Prevention Network (WVAATPN) in 2021. The WVAATPN effectively extended prevention and cessation efforts to grassroots levels, including places of worship and homes, by assembling a network of faith and community-based lay helpers to educate their respective communities about the perils of tobacco use. The Network educated these lay helpers so they could then provide educational and cessation workshops to members of their communities.

National partners and experts provided crucial technical assistance to the Network, enhancing their proficiency in African American Tobacco Prevention and Menthol Control. Notable contributions came from the “Not in Mama’s Kitchen” education campaign led by Brenda Bell.

Caffee (24), the “Same Game, Different Smokers” curriculum endorsed by the African American.

Tobacco Control Leadership Council (chaired by Phillip Gardiner) (25, 26), and La Tanisha Wright’s “Follow the Signs” counter-marketing training (27). These experts assisted in developing the evidence-based curriculum to train the lay helpers.

## 3 Learning environment

The Network was formed by churches and other community organizations across West Virginia. Churches were identified based upon their location and willingness to participate. The locations were selected by focusing on the top 10 communities within West Virginia with the highest rates of African Americans within the current population. Willingness was defined as having the pastor agree for the congregation to participate and the willingness to have a lay helper be trained to implement the program. The Network’s membership encompasses various congregations across counties, including Raleigh, Kanawha, Fayette, Mercer, Greenbrier, Cabell, and McDowell. The number of participating congregations has grown significantly, from 7 in 2021–2022 to 14 in 2022–2023, and currently stands at 22 (28).

This congregation-based approach allows for direct community involvement instead of distant, state-based efforts. The local nature of these efforts means congregations can personalize the messages for their community and communicate directly to those who need to hear those messages the most. Those who need the support are often found within the congregations and are the first targets of recruitment for the cessation and education workshops. As needed, congregation leaders can then move their recruitment efforts beyond their members into the broader community. Congregation leaders target adults for the workshop, not youth. The state-wide, youth anti-tobacco program known as RAZE is already actively targeting and reaching youth (29).

Additionally, the Network established an advisory board to provide valuable guidance and direction for its initiatives, ensuring effective decision-making and strategic planning. Board Members were selected by identifying community champions from sectors needed to give validity to the network, including government, education, faith leaders, and advocacy networks. The creation of the advisory board was a necessary step to ensure the longevity and continued success of the Network.

To broaden its influence, the Network has forged strategic partnerships with key organizations such as the Center for Black Health and Equity, the West Virginia Division of Tobacco Prevention, and the Claude Worthington Benedum Foundation. These collaborations have enabled the Network to diversify its funding sources and have provided a platform to exchange knowledge with other networks nationwide. Additionally, they have facilitated partnerships with regional and national agencies, enhancing their collective efforts. It is also vital to note that the Benedum Foundation partnership has brought the Network into contact with other agencies focused on serving the African American community throughout West Virginia and brought much-needed validity to the Network in the eyes of community leaders. The Center for Black Health and Equity has put the Network on a national platform to tell its story and helped to bring national-level experts to train the Network staff and congregation lay helpers.

### 3.1 Pedagogical format

Each fall, the Network convenes its congregational members and appoints lay helpers to participate in training sessions led by state and national experts. These lay helpers have the responsibility to run workshops designed to help people become educated about tobacco use and have the resources they need to quit. It is essential that these lay helpers be well trained on these topics so that they can then share their knowledge when they run the workshops. These topics are often complex, requiring the lay helpers to have a strong understanding of them to be able to teach them. Additionally, adult learners need to know why they need to learn something before learning it (30), so lay helpers are trained to teach the importance of these principles as they teach them. Topics covered include Menthol and African Americans, predatory marketing tactics of tobacco companies, Quitline services, secondhand smoke education, clean indoor air regulations, youth tobacco prevention, conducting tobacco cessation workshops, and workshop evaluation methods. Following these sessions, each congregation develops annual educational plans. Additionally, congregations have dedicated staff available for assistance, including access to a Certified Tobacco Treatment Specialist.

The Network’s model consists of multiple congregations across counties. In each county, lay helpers focus on education and cessation. This includes educational workshops to teach about the impact of tobacco use and other events like No Menthol Sunday. These workshops, hosted at individual churches, provide community members with an opportunity to learn about tobacco at a site close to home.

#### 3.1.1 Tobacco education workshops

The education sessions focus on the dangers of tobacco use, secondhand smoke, menthol, and the importance of cessation. All participants complete the same pre-and post-evaluation surveys for the purpose of program evaluation and improvement. This workshop is taught from a PowerPoint developed by Rhonda Robinson from the WVAATPN. Some of the learning objectives of the workshop include the following:

- Understanding the tobacco use rates in the United States and West Virginia.
- Understanding the patterns of tobacco use for African Americans.
- Understanding tobacco industry marketing and influence within the African American community.
- Understanding the dangers of exposure to secondhand smoke.
- Understanding how tobacco use affects the body.
- Understanding the West Virginia Tobacco Quitline services.

#### 3.1.2 Tobacco cessation workshops

The Network also sponsored tobacco cessation workshops to help individuals take the necessary steps to quit. Lay helpers use the Pathways to Freedom Guidebook and Video for the cessation workshops. The Pathways to Freedom Guidebook is a free resource and can be accessed online at https://stacks.cdc.gov/view/cdc/11397. All participants complete the same pre-and post-evaluation surveys for the purpose of program evaluation and improvement. Some of the learning objectives of the workshop include the following:

- Understanding nicotine addiction.
- Understanding the steps to quitting.
- Understanding how the body responds to nicotine withdrawal.
- Understanding what medications can help someone quit.
- Understanding how to deal with the stress of quitting.
- Understanding the West Virginia Tobacco Quitline services.

As part of the Network’s focus on African Americans, the West Virginia Tobacco Quitline incorporated the same Pathways to Freedom Guidebook and Video used in the cessation workshops for all African Americans enrolled in cessation services. This was a significant cultural competence strategy for the Network.

## 4 Results

Though the Network is new (founded in 2021), it has already had a powerful impact across West Virginia. Its grassroots approach to tobacco education and cessation has increased awareness about the issue and motivated people to give up tobacco for good. Specifically, the Network quickly organized the African American Community and mobilized lay helpers by providing essential and effective training. This has resulted in increased knowledge about the dangers of tobacco use and increased cessation attempts. Most importantly, it has brought together local, state, and national organizations to overcome this deep-rooted West Virginia challenge.

### 4.1 Tobacco cessation workshops

In total, 212 individuals participated in the 22 tobacco cessation workshops across the state. While we did not record demographic data of the participants, from photo observations it appears that they were approximately 40% male and 60% female. Of the participants, 75% (160) completed the pre-and post-training surveys. Of the participants, 57% had used tobacco for more than 5 years. Their tobacco product use varied, with 61% using Cigarettes, 18% using E-Cigarettes/Vapes, and 12% using Cigars. In addition, 75% of them had a previous quit attempt.

Some of the notable improvements from the workshop post-evaluation surveys included an 18% increase on a weighted Likert scale in participants’ responses to the statement, “I know about medicines that can help me quit” and a 16% increase on a weighted Likert scale in participants’ response to the statement, “I understand how the body responds to nicotine withdrawal” (Table 1). This increase in understanding about quit medicines and the effects of withdrawals is essential in helping individuals to be motivated to quit.

**Table 1:** The self-reported program evaluation responses to the questions posed during the cessation workshops using a weighted Likert scale.

Question	Pre-training survey	Post-training survey	Change
I want to quit using tobacco.	3.86	4.19	+8.5%
I understand nicotine addiction	4.31	4.49	+4.17%
I understand the steps to quitting	3.91	4.41	+12.78%
I understand how the body responds to nicotine withdrawal.	3.74	4.37	+16.84%
I know about medicines that can help me quit.	3.62	4.28	+18.23%
I am confident I can stop using tobacco.	3.77	4.01	+6.36%
I am confident I can control my urges to use tobacco.	3.54	4.01	+13.27%
I know how to deal with the stress of quitting tobacco.	3.55	3.99	+12.39%
My friends and family will support my efforts to quit.	4.24	4.30	+1.4%

It highlights the pre-training responses, post-training responses, and the percent change. *N* = 160 (75% of Participants).

After the workshop, all participants who supplied an email or phone number received the follow-up survey. Those who provided an email were contacted twice. The follow-up survey had a 23% response rate, which is within the typical range. Of those who responded, 37% reported calling the West Virginia Tobacco Quitline after the Workshop; 16% reported quitting during the project period, and of those still using tobacco, 71% reported seriously thinking about quitting within the next 6 months. We are working on strategies to increase the follow-up survey response rate.

### 4.2 Tobacco education workshops

In total, 286 people participated in the 21 tobacco education workshops across the state. While we did not record demographic data of the participants, from photo observations it appears that they were approximately 40% male and 60% female. Of the participants, 77% (220) completed the pre-and post-training surveys. Among the attendees, 25% had used tobacco for more than 5 years. The most common form of tobacco product was cigarettes, and 25% of homes had a tobacco user in them. Of those who used tobacco, 36% had previously tried to quit.

Some of the notable improvements from the workshop post-evaluation surveys included a 25% increase on a weighted Likert scale in participants’ response to the statement, “I can explain the WV Tobacco Quitline Services” and a 13% increase on a weighted Likert scale in participants’ response to the statement, “I understand marketing strategies used in African American targeted advertising” (Table 2). This was an important result because the West Virginia Tobacco Quitline is an invaluable resource in helping people quit, and increased understanding of its services means people are more likely to call. Additionally, an increased knowledge of targeted advertising to African Americans helps people be more aware of and resistant to advertising.

**Table 2:** The self-reported program evaluation responses to the questions posed during the education workshops using a weighted Likert scale.

Question	Pre-training survey	Post-training survey	Change
I understand the tobacco use prevalence rates among African Americans in the USA and WV.	3.98	4.34	+9%
I understand the patterns of tobacco use.	3.93	4.36	+11%
I understand the health effects of tobacco use.	4.24	4.52	+6.6%
I understand the dangers of secondhand smoke.	4.19	4.50	+7.39%
I understand marketing strategies used in African American targeted advertising.	3.91	4.44	+13.55%
I can explain the WV Tobacco Quitline Services.	3.39	4.25	+25.36%

It highlights the pre-training responses, post-training responses, and the percent change. *N* = 220 (77% of participants).

### 4.3 No Menthol Sunday

No Menthol Sunday is an annual call to action started 10 years ago by the Center for Black Health and Equity. The Network was instrumental in guiding and implementing the 2023 and 2024 No Menthol Sunday Campaign in West Virginia, effectively raising awareness about the dangers of mentholated tobacco products. By mobilizing the 22 congregations who participated and leveraging the power of social media, the campaign reached a staggering 22,000 West Virginia citizens. The network members implemented numerous strategies such as placing the Quitline information in church bulletins, creating church display boards, displaying No Menthol Banners, including information in the sermons, and hosting information sessions on No Menthol Sunday.

### 4.4 Quitline calls July 2023–May 2024

There were 144 Quitline calls across the state from July 2023 to May 2024. This is a 61% increase from the prior year. As a matter of protocol from this project, all African American enrollees were given the Pathways to Freedom Guidebook and Video by the Quitline. This is a key culturally competent strategy that was added due to the influence of the Network.

## 5 Lessons learned and suggestions for future projects

Forming a faith-based network of African American churches to unite in the fight against tobacco is not a new concept. One similar study involved recruiting churches through contact with pastors, surveying the community to understand baseline smoking rates, training lay helpers to conduct health exams and teach classes, developing and distributing materials, and planning worship services centered around overcoming tobacco. These lay helpers then administered cessation lessons to congregation members willing to participate. Additionally, they established an advisory board to guide the actions of the organization (31). In developing the WVAATPN, we followed a similar pattern, with a key difference being that we did not survey the community beforehand to understand the patterns of tobacco use, but instead focused our efforts on the cessation and education workshops. Additionally, we did not perform health screenings as part of this project.

The efforts of the WVAATPN focused on helping African Americans become educated about tobacco use and empowered to overcome it. Because the Network focused on a specific demographic, the lay helpers were able to successfully tailor their efforts to the specific needs of that community. The education and cessation workshops focused on the unique needs of African Americans and were more culturally relevant than general principles would be. For example, focusing on the tobacco industry’s targeted marketing to African Americans made the workshops personally relevant to participants. Additionally, because the Network utilized lay helpers from various communities across West Virginia, they were more responsible for the outcomes of the workshops. Rather than sending workers to other communities, where they would have little familiarity, the lay helpers became the experts for their area and had more credibility among the participants due to their locality. As these lay helpers conducted their workshops, they made them interactive, allowing participants to learn in a more dynamic environment than if they had solely been given a handbook to read. The workshops included discussions, time for questions, and activities that increased understanding and solidified the concepts being taught. To give background to this challenge, time was taken in the workshops to speak about how African Americans are disproportionately affected by tobacco use (22, 23). Creating an open dialogue about these challenges and their impact on the African American community made participants more aware of these challenges and more motivated to address them. Additionally, both the tobacco education and cessation workshops focused on actionable objectives designed to increase participants’ understanding of tobacco use and their ability to overcome tobacco addiction. By focusing on actionable objectives of increasing understanding and core competencies, lay leaders helped the participants to not just understand how to quit, but also have the skills necessary to do so. The workshops gave participants the opportunity to develop actionable skills necessary to conquer tobacco.

As we look back on this project, we learn many clear lessons about tobacco use and its prevention. This also leads us to policy recommendations that will help the West Virginia African American community and others to increase the effectiveness of their efforts to overcome tobacco use. To begin, in general, as poverty increases, so does the prevalence of smoking (32). The high prevalence of tobacco use in West Virginia is not an exception, as nearly 18% of the population live in poverty (4). This contributes to a cycle where limited financial resources are further strained by tobacco expenditure, exacerbating health disparities (6). Effective tobacco prevention programs must address underlying socio-economic issues to break this cycle. One policy change that helps address this is a tobacco tax, which has been shown to reduce smoking in those with low socioeconomic status (33). A tobacco tax raises tobacco prices, which gives those in poverty high motivation to quit, including youth, because their tobacco habits are not as established (34, 35).

Another challenge that confronts West Virginia is the absence of state-level Clean Indoor Air Regulations, increasing tobacco-related health issues in both smokers and nonsmokers (2). Local regulations are often preemptively challenged, weakening the overall impact of tobacco control efforts (3). We advocate for establishing and enforcing comprehensive state-level policies because they are crucial for reducing exposure to secondhand smoke and lowering smoking rates (3). In addition to a lack of state-level Clean Indoor Air Regulations, African American communities in West Virginia are disproportionately targeted by tobacco companies through menthol cigarette marketing (15–17). This targeted approach has led to higher smoking rates and related health disparities in these communities (22, 23). A major policy needed to overcome these challenges is a ban on menthol cigarettes, which could help more smokers to quit (36).

Finally, despite receiving significant funds from the Master Settlement Agreement and tobacco taxes, West Virginia ranks lowest in the U.S. for tobacco prevention spending (1). Adequate funding is essential for the success of prevention programs. Increased investment in tobacco prevention can lead to better health outcomes and reduced healthcare costs in the long term (37). Undeterred by low funding, the West Virginia African American Tobacco Prevention Network (WVAATPN) has demonstrated the effectiveness of community-led initiatives. By mobilizing local organizations and forming strategic partnerships, WVAATPN has increased awareness, improved cessation rates, and enhanced understanding of tobacco’s health impacts. Engaging community leaders and collaborating with national experts are key components for the success of these initiatives.

## 6 Limitations

### 6.1 Data collection limitations

The data used in analyzing tobacco use and its impact in West Virginia rely on self-reported program evaluation surveys and statistical estimates, which May be subject to reporting biases and inaccuracies. Additionally, the availability of up-to-date and comprehensive data can be limited, potentially affecting the precision of the findings.

### 6.2 Generalizability of findings

The intent of this work was not to generalize knowledge or test hypotheses. The intent of this program evaluation was to improve the education and cessation workshops conducted by the West Virginia African American Tobacco Prevention Network and to increase calls to the WV Tobacco Quitline.

### 6.3 Scope of socio-economic factors

The analysis primarily focuses on poverty as a critical socio-economic factor influencing tobacco use. However, other factors such as education, employment, access to healthcare, and social support systems also play significant roles and May not be fully accounted for in the program evaluation results.

### 6.4 Measurement of program impact

Assessing the impact of tobacco prevention programs, such as those led by WVAATPN, can be challenging due to the difficulty in isolating the effects of specific interventions from other influencing factors. Longitudinal evaluation and more rigorous evaluation methods are needed to measure program effectiveness and long-term outcomes.

### 6.5 Marketing influence on targeted populations

The program identifies the targeted marketing of menthol cigarettes to African American communities as a significant issue. However, the extent of the influence of these marketing tactics and the specific pathways through which they affect smoking behavior requires further detailed investigation.

### 6.6 Resource allocation and funding challenges

Underfunding of tobacco prevention efforts in West Virginia is a critical constraint. While the program highlights this issue, it May not fully capture the complexities of budget allocations, political priorities, and competing public health needs that influence funding decisions.

## Data Availability

The original contributions presented in the study are included in the article/supplementary material, further inquiries can be directed to the corresponding author.
